# Petals Reduce Attachment of Insect Pollinators: A Case Study of the Plant *Dahlia pinnata* and the Fly *Eristalis tenax*

**DOI:** 10.3390/insects14030285

**Published:** 2023-03-14

**Authors:** Elena V. Gorb, Stanislav N. Gorb

**Affiliations:** Department of Functional Morphology and Biomechanics, Zoological Institute, Kiel University, Am Botanischen Garten 9, 24098 Kiel, Germany

**Keywords:** attachment force, “cafeteria”-type flowers, cuticular folds, epicuticular wax projections, flower stems, leaves, pollination, papillae

## Abstract

**Simple Summary:**

One of the important aspects in the relationship between plants and insects is pollination including attraction of pollinators, which is related to mainly optical effects. Since insects pollinating the majority of plants are in contact with a petal surface for a certain time, it is plausible to hypothesize that such petals should also enable or even promote attachment of insects to their surface. The aim of this study was to understand whether the petal surface in “cafeteria”-type flowers, which offer their nectar and pollen to insect pollinators in an open way, is adapted to a stronger attachment of pollinators. We selected the garden dahlia plant *Dahlia pinnata* and the hovering fly *Eristalis tenax,* examined leaves, petals, and flower stems using cryo scanning electron microscopy, and performed force measurements of fly attachment to surfaces of these plant organs. Our experimental results showed that insect attachment on the petal surface was significantly weaker compared to that on smooth leaf and smooth reference glass. In our opinion, these “cafeteria”-type flowers have the petals, where the colour intensity is enhanced due to papillate/conical epidermal cells covered by micro- and nanoscopic cuticular folds, and exactly these latter structures mainly contribute to attachment reduction in insect pollinators.

**Abstract:**

In order to understand whether the petal surface in “cafeteria”-type flowers, which offer their nectar and pollen to insect pollinators in an open way, is adapted to a stronger attachment of insect pollinators, we selected the plant *Dahlia pinnata* and the hovering fly *Eristalis tenax*, both being generalist species according to their pollinator’s spectrum and diet, respectively. We combined cryo scanning electron microscopy examination of leaves, petals, and flower stems with force measurements of fly attachment to surfaces of these plant organs. Our results clearly distinguished two groups among tested surfaces: (1) the smooth leaf and reference smooth glass ensured a rather high attachment force of the fly; (2) the flower stem and petal significantly reduced it. The attachment force reduction on flower stems and petals is caused by different structural effects. In the first case, it is a combination of ridged topography and three-dimensional wax projections, whereas the papillate petal surface is supplemented by cuticular folds. In our opinion, these “cafeteria”-type flowers have the petals, where the colour intensity is enhanced due to papillate epidermal cells covered by cuticular folds at the micro- and nanoscale, and exactly these latter structures mainly contribute to adhesion reduction in generalist insect pollinators.

## 1. Introduction

One of the evolutionary pressures that has led to rise and development of a great variety of surface micro-and nanostructures in plants is a co-evolution between plants and insects [1,2,3]. Thus, herbivory has resulted in an appearance of features and adaptations associated with plant defence against herbivorous insects. An important role of plant trichomes (hair-like protuberances extending from the epidermis of aerial plant tissues), covering leaves against herbivores, is well known [1,2,4]. For example, hooked trichomes of *Phaseolus* plants (Fabaceae) are able to entrap and kill a number of insect species belonging to different insect orders, such as Hemiptera and Diptera [5,6]. Effects of plant pubescence differ according to the insect herbivore species [1,7] as well as the trichome type (non-glandular or glandular), size, and distribution [8]. Glandular trichomes may additionally produce sticky (e.g.,*Datura*, *Lycopersicon*, *Nicotiana*, and *Solanum* species from the Solanaceae family) [9] or even poisonous secretions, which provide resistance against insect pests [10,11]. Additionally, microscopic surface features, such as cuticular folds (cuticle sculpturing of the plant surface usually caused by folding of the cuticle over the outer cell wall of epidermal cells), can deter herbivores, presumably due to reduction in insect attachment caused by minimization of the real contact area between insect attachment organs and microrough plant surface. This effect has been experimentally shown for petals and leaves of several plant species and the beetle *Leptinotarsa decemlineata* (Coleoptera: Chrysomelidae) [12,13,14]. Three-dimensional projections of epicuticular wax (a complex mixture of long-chain aliphatic and cyclic hydrocarbons, fatty acids, aldehydes, b-diketones, primary and secondary alcohols deposited onto the plant surface) composing a pruinose microrough coverage on aerial primary surfaces of higher plants also greatly contribute to plant protection against herbivorous and phytophagous insects. The protective function has been repeatedly reported and experimentally supported for numerous plants, for example *Eucalyptus* (Myrtaceae), *Pisum* (Fabaceae), and *Brassica* (Brassicaceae) species (see review by Gorb and Gorb [15]). These micro-/nanoscopic surface structures reduce the attachment ability of insects and impair their locomotion performance, possibly due to (1) the reduction in the real contact area between the tips of insect attachment organs (pads) and the plant surface (the roughness hypothesis); (2) the contamination of insect adhesive organs by plant wax (the contamination hypothesis); (3) the absorption of fluid from the insect adhesive pads due to the high capillarity of the wax coverage (the fluid absorption hypothesis); (4) hydroplaning caused by the appearance of a thick layer of liquid caused by the dissolving of the wax in insect adhesive fluid (the wax dissolving hypothesis); and (5) the formation of a separation layer between the plant substrate and insect attachment organs [16,17].

On the other hand, carnivorous plants, which rely on insect prey, have evolved specialized trapping organs bearing various surface structures, in order to capture and retain insects and other small animals for additional nutrition. For instance, in representatives of the plant genera *Gensilea* (Lentibulariaceae) with lobster-pot traps and *Cephalotus*, *Darlingtonia*, *Heliamphora*, and *Sarracenia* (all Sarraceniaceae) with pitfall traps, long, often sharp, downward- or inward-pointing trichomes allow an animal’s progress into the trap, but prevent its moving into the opposite direction [2,18]. Movable glandular trichomes covering leaves in *Drosera* species (Droseraceae) (flypaper traps) use sticky mucilage to impede and immobilize prey animals [18]. Three-dimensional epicuticular wax coverages on the inner surface of pitchers in carnivorous plants from the genera *Nepenthes* (Nepentheceae), *Sarracenia*, and *Darlingtonia*, and in certain insect-trapping Bromeliaceae, contribute to prey trapping and retention [18,19,20,21,22].

Another important aspect of relationships between plants and insects is pollination, including attraction of pollinators, which is related to mainly optical effects, primarily generation and amplification of colour intensity or chemical attributes [22,23,24], or to other specialized mechanisms developed in kettle trap flowers. For pollination purposes, pitfall traps have been developed in representatives of the plant families Araceae, Aristolochiaceae, Apocynaceae s.l., Hydnoraceae, and Orchidaceae [25]. In plants from the genera *Aristolochia* (Aristolochiaceae) and *Ceropegia* (Apocynaceae), flowers are equipped with specialized trapping trichomes showing similar effects on pollinating insects during capture and retention stages as those in carnivorous pitfall traps [25,26,27]. Certain plants with kettle trap flowers, for example *Aristolochia* and *Arisaema* (Araceae) species, bear slippery wax-covered surfaces contributing to temporary capture of their pollinators [25,28,29].

Since insects pollinating the majority of plants are in contact with a petal surface for a certain time, it is plausible to hypothesize that such petals should enable or even promote attachment of insects to their surface. It is known that petals of many insect-pollinating flowers have papillate or cone-shaped epidermal cells/structures [30,31,32,33,34,35] mainly serving colour intensity enhancements in different directions that result in an increase in flower perceptibility both from a long distance and under different angles of view.The mechanical effects of such petal surface characteristics on the attachment of insect-pollinators still remain rather poorly understood. Thus, the contribution of petal surfaces to their mechanical stability that ensures the interlocking of flower visitors (insects) with well-developed claws to the conical epidermal cells and/or papillae has been reported on [31,32,33,35]. On the contrary, viscous or oily coverage as well as epicuticular wax projections, present on particular petal sites in some flowers, may prevent attachments of any insect [24,36]. Experimental study on attachment ability of the honeybee *Apis mellifera carnica* Pollmann (Hymenoptera, Apidae) and greenbottle fly *Lucilia caesar* L. (Diptera, Calliphoridae) to petals of ten plant species differing in their surface texture showed that both insect pollinators had a good foothold on rough surfaces including conical and papillate epidermal cells, while flat epidermal cells with microstructures such as cuticular folds and wax projections significantly reduced insect attachment [37].

However, taking into account the diversity of petal microstructures shown in the above studies, it is difficult to judge the particular surface adaptations of petals without comparing them to other surfaces in the same plant. For this study, we selected an insect-pollinating species *Dahlia pinnata* Cav. (Asteraceae) having distinctly different surfaces of leaves vs. petals vs. stems and a hovering fly pollinator *Eristalis tenax* (L.) (Diptera: Syrphidae). Both the fly and plant are generalist (not specialist) species in terms of their diet and pollinators spectrum, respectively. This plant is usually visited by representatives of various insect groups including bees, bumblebees, flies, etc. Adults of *E. tenax* as well as those in the majority of syrphid flies feed on pollen of numerous plants from different families (Asteraceae, Apiaceae, Adoxaceae, etc.), which offer their nectar and pollen in an open way (“cafeteria”-type of flowers). The study is based on a combination of cryo scanning electron microscopy (cryo-SEM) examination of related plant and insect surfaces and force measurements of the attachment of *E. tenax* to surfaces of different plant organs. Our aim was to find out whether the petal surface is adapted to a stronger attachment of the insect. For comparison, we used leaf and flower stem surfaces of *D. pinnata*.

## 2. Materials and Methods

### 2.1. Plants and Insects

**Figure 1:**
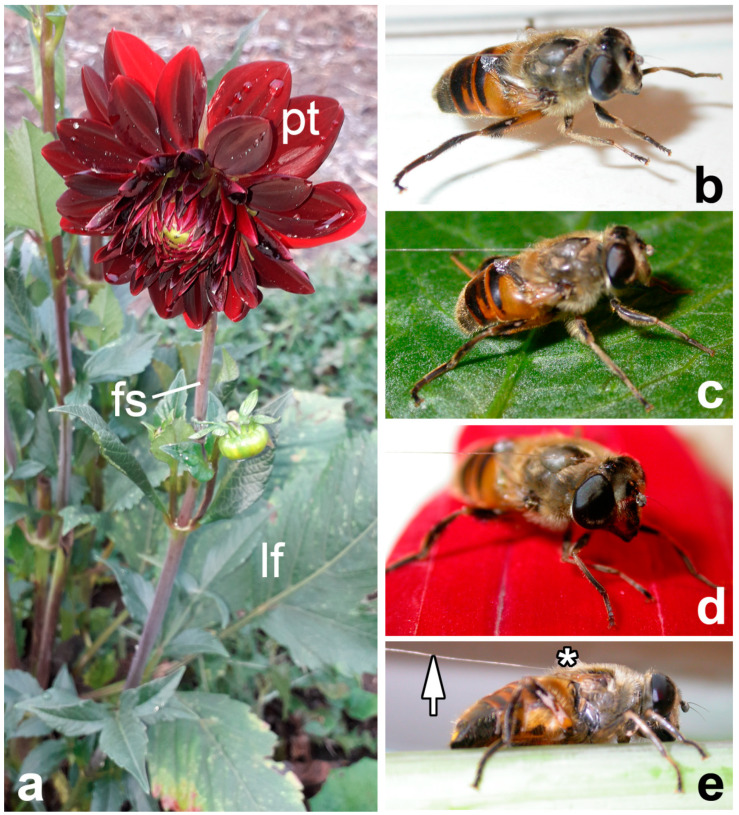
The plant *Dahlia pinnata* (**a**) and the fly *Eristalis tenax* in the experiments on different test substrates (**b**–**e**): glass (**b**), the adaxial (upper) side of the leaf (**c**), the adaxial side of the petal (**d**), and the stem (**e**). In (**e**), arrow points to a hair attached dorsally to the fly thorax using a droplet of molten beewax (asterisk). Abbreviations: fs, flower stem; lf, upper leaf; pt, petal (actually, ray flower).

The garden dahlia *D. pinnata* (Figure 1a) is a 30–200 cm high perennial herbaceous plant with erect stems branching only in the inflorescence, usually simple ovate leaves, and 2–8 flower stems later called “stems” (5–15 cm long) each bearing a flower head (capitulum) with both tubular and ovate strap-shaped florets (ray flowers or ligulate flowers). As a ligula represents fused petals, we called it “petal” throughout the text. Being native to Mexico, Central America and Colombia, *D. pinnata* is now extensively cultivated worldwide as a decorative plant [38]. Plant samples (stems, upper leaves, and petals) for cryo-SEM examination and experiments (Figure 1) were collected from garden plants (Jagotyn, Kiev District, Ukraine, 50°17’ N 31°46’ E).

**Figure 2:**
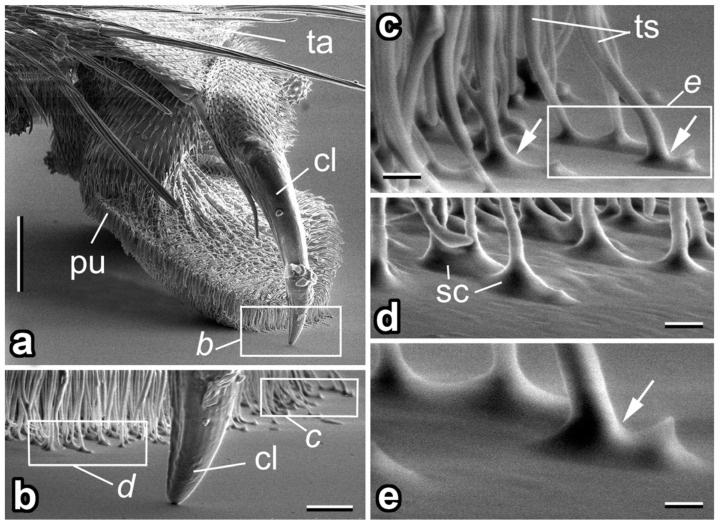
The attachment system of the fly *Eristalis tenax* (cryo-SEM). (**a**) Dorso-lateral view of the distal part of the tarsus (ta) with pretarsus bearing claws (cl) and pulvilli (pu). (**b**) The claw (cl) and distal portion of the pulvillus in contact with a smooth substrate. (**c**–**e**) Tenent setae (ts) covering the pulvillus in contact with the substrate. Note the pad secretion (sc) delivered into a contact zone. Arrows in (**c**,**e**) show examples of the setal terminal elements, spatulae, in contact. Scale bars: 100 µm (**a**), 10 µm (**b**), 2 µm (**c**,**d**), 1 µm (**e**).

The common drone fly *E. tenax* mimicks a stock bee [39] that feeds on the nectar and pollen of flowers [40,41]. It has a cosmopolitan distribution and is the most widely distributed syrphid species in the world [42]. Insects were captured in August 2005 near Jagotyn and used for structural examination and experiments. The attachment system of *E. tenax* is composed of paired claws and paired setose pulvilli on each foot (Figure 2a,b) (for details see [43]). Microscopic tenent setae covering the pulvilli have spatula-shaped terminal elements (Figure 2d) responsible for building intimate contact with a substrate (Figure 2b–e). Additionally, a pad fluid is secreted into a contact zone (Figure 2c–e).

### 2.2. Microscopy

Plant samples (stem, upper leaf, and petal) (Figure 1a) for cryo-SEM examination were cut off from living plants and kept for 24 h inside small plastic vials containing wet paper in order to prevent desiccation of the plant material. Next, small (1 cm × 1 cm) portions were cut out from the middle regions of the leaf and petal and from the stem, glued with polyvinyl alcohol Tissue-Teck O.C.T.TM Compound (Sakura Finetek Europe B.V., Zoeterwoude, The Netherlands) to a metal holder, and frozen in a cryo stage preparation chamber at −140 °C (Gatan ALTO 2500 cryo preparation system, Gatan Inc., Abingdon, UK). Frozen samples of the upper (adaxial) side, in both the leaf and petal, and the stem surface were sputter coated with gold–palladium (6 nm thickness) and studied in a frozen condition in a cryo-SEM Hitachi S-4800 (Hitachi High-Technologies Corporation, Tokyo, Japan) at 3 kV accelerating voltage and −120 °C temperature. Microscopy was performed at the Max Planck Institute for Metals Research (Stuttgart, Germany).

Types of wax projections were identified according to [44]. Morphometrical variables of plant surface features were measured from digital images using the software SigmaScan Pro 5 (SPSS Inc., Chicago, IL, USA). These data are given in the text as mean ± SD for n = 10.

### 2.3. Experiments

Force measurements were carried out using a load cell force sensor FORT-10 (10 g capacity; World Precision Instruments Inc., Sarasota, FL, USA) connected to a force transducer MP 100 (Biopac Systems Ltd., Santa Barbara, CA, USA) [45,46]. Prior to the experiments, test insects were made incapable of flying by cutting off their wings. After a certain recovery time, the fly was attached to the force sensor by means of a 10–15 cm long hair glued to the dorsal side of its thorax with a droplet of molten beeswax (Figure 1e). The force produced by the insect walking horizontally on the test substrates and pulling the hair (Figure 1b–e) for approximately 5–30 s was measured. Force–time curves were used to estimate the maximal friction (traction) force produced by insects. Since the flies were constrained to pulling parallel (not at an angle) to the measurement axis of the sensor, the registered force corresponded to the total traction force. Three types of fresh plant samples were used as substrates: (i) the adaxial side of the upper leaf; (ii) the adaxial side of the petal; and (iii) the stem, where insects move towards the apical stem direction (Figure 1c–e). A smooth hydrophilic glass surface served as a control (Figure 1b). For each insect individual, the test substrates were used in the following sequence: (1) glass as a reference, (2 or 3) leaf, (3 or 2) petal, and (4) stem. Leaf and petal samples were randomized in the consecutive test series. Taking into account that the stem surface is covered with three-dimensional epicuticular wax projections [47], we performed experiments on the stem surface at the end of a test series, in order to avoid the effect of a possible contamination of insect feet by wax particles on the experimental results.

Force tests were carried out at 22–25 °C and ca. 60–75% humidity. On each substrate type, experiments with 10 individual flies (10 repetitions per insect) were performed. In all, 400 force tests were conducted.

Force values were analysed using two-way analysis of variance (ANOVA), considering the insect individual and the substrate as factors (software SigmaPlot 11.0, SPSS Inc., Chicago, IL, USA).

## 3. Results

### 3.1. Micromorphology of Dahlia pinnata Surfaces

**Figure 3:**
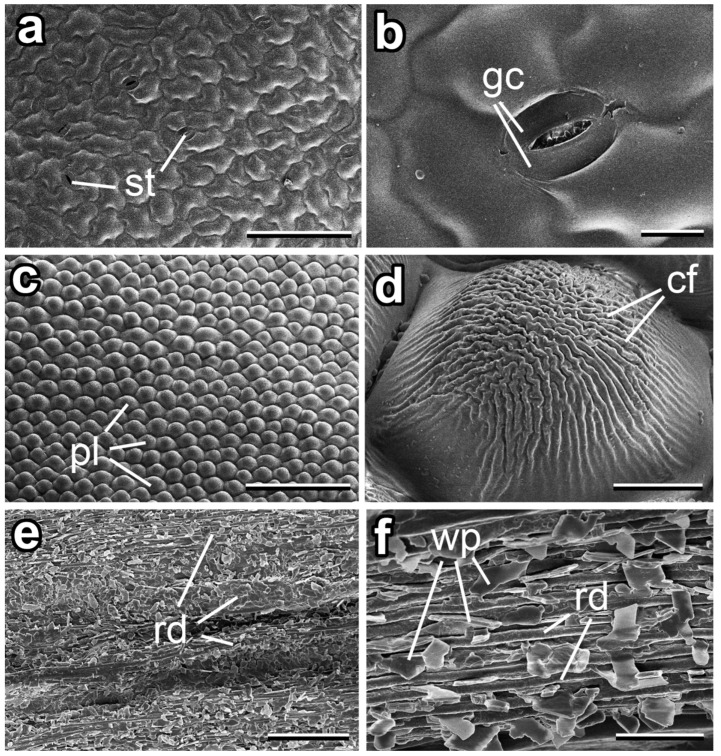
Surfaces of different organs in the plant *Dahlia pinnata* (cryo-SEM). (**a**,**b**) Adaxial leaf side. (**c**,**d**) Adaxial petal side. (**e**,**f**). Stem. Abbreviations: cf, cuticular folds; gc, guard cells of the stoma; pl, papillae; rd, ridges; st, stomata; wp, epicuticular wax plates. Scale bars: 200 µm (**a**,**c**), 20 µm (**b**,**e**), 10 µm (**d**), 5 µm (**f**).

The adaxial leaf side was slightly uneven because of epidermal cells showing a somewhat convex shape of the outer walls and of the sparsely dispersed (abundance: ca. 0.15 mm^−2^) stomata, having slightly sunk guard cells (Figure 3a,b). The surface had a smooth appearance (Figure 3b).

The adaxial petal surface was strongly uneven (Figure 3c). It was characterized by the papillate, polygonal epidermal cells, which were noticeably structured with rather uniform cuticular folds (width: 1.68 ± 0.34 µm; height: 0.68 ± 0.16 µm) (Figure 3c,d). Originating at the cell periphery, at first the folds were straight and rather low, becoming more and more wavy, higher (height: 0.69 ± 0.11 µm), and densely spaced (distance between folds: 0.51 ± 0.08 µm) to the centre of the papillae (Figure 3d).

Stems of *D. pinnata* had a hierarchically arranged system of ridges and grooves (Figure 3e,f). The surface bore a three-dimensional epicuticular coverage consisting of separate wax plates (abundance: ca. 0.3–0.5 plates per 1 µm) (Figure 3f). The plates varied both in dimensions (length: 2.58 ± 0.67 µm; width: 1.46 ± 0.38 µm; thickness: 0.19 ± 0.06 µm) and shapes, however, being more often rhomb shaped. Their edges were usually rather distinct, but also projections with slightly irregular edges occurred. The plates showed no preferred orientation. They protruded from the cuticle surface at various angles and were attached to the latter either by their narrow or larger sides. Such a variation in plate connections to the underlying surface was possibly caused by the particular (hierarchical ridges/grooves) geometry of the stem.

### 3.2. Traction Forces on Different Test Substrates

**Figure 4:**
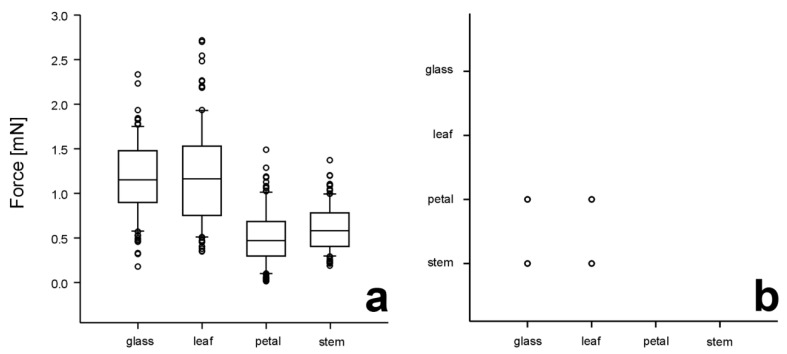
Traction forces produced by flies *Eristalis tenax* on different substrates (glass, adaxial sides of the upper leaf and the petal, and the stem in the plant *Dahlia pinnata*) (**a**) and results of the statistical comparison of the force values obtained on different substrates (**b**). In (**a**), boxplots show the interquartile range and the medians, whiskers indicate the 1.5 × interquartile range, and “°” are outliers. In (**b**), circles mean significant differences in force values between substrates at *p* < 0.05 (Tukey post hoc test, two-way ANOVA).

Traction force values of 10 individuals of *E. tenax* obtained on glass and different plant surfaces are presented in Figure 4a. Since the force generated by different insect individuals varied greatly even on glass (1.18 ± 0.38 mN, n = 100), we analysed the obtained data using two-way ANOVA, considering both the insect individual and the substrate as factors. The flies showed significantly different forces (*p* < 0.001, two-way ANOVA). Both factors, insect individuals and substrates, significantly influenced the force values (individuals: F*_9,391_* = 37.565, substrates: F*_3,397_* = 213.416, both *p* < 0.001). The similarly higher forces were registered on reference glass and the leaf surface (Figure 4). These force values significantly differed from much lower ones obtained on petals and stems, which did not differ significantly from each other (Figure 4).

## 4. Discussion

Our experimental results clearly distinguished two groups among tested surfaces. Reference glass and the upper leaf surface ensured rather high attachment force of the *E. tenax* fly, whereas both the flower stem and petal significantly reduced it. Strong attachment on smooth, both artificial and natural, surfaces has been observed in many insect species for a rather long time [16,46,48,49,50,51]. However, the attachment force reduction on flower stems and petals is caused by different structural effects. In the case of the flower stem, it is the combination of ridged topography and three-dimensional wax projections [47], whereas the papillate petal surface is supplemented by cuticular folds. The effect of the three-dimensional wax coverage on insect attachment has been repeatedly reported in numerous studies (reviewed in [15]) and several contributing mechanisms, such as surface roughness, insect pad contamination, pad fluid absorption, hydroplaning, and separation layer, have been proposed [16,17] and, in part, experimentally proved [21,46,52,53,54]. The role of cuticular folds in a decrease in insect attachment force has also been recently revealed by several experimental studies [12,13,14].

The biological reason of insect attachment force reduction on the flower stem is rather clear and is associated with a greasy pole syndrome, i.e., a plant defence mechanism preventing ants from visiting flowers and robbing nectar [55,56,57]. It has been described in *Salix* (Salicaceae*), Hypenia,* and *Eriop*e (both Lamiaceae) plant species and is based on the combined effect of several stem features, such as slender elongate erect stems, rigid spreading trichomes on lower internodes, three-dimensional wax coverage, and, often, swellings in upper internodes, which hamper access of ants to apically located plant reproductive organs [57]. Recently, this syndrome was experimentally studied in several other plants [47,58,59], *D. pinnata* among them [47], where main attention was given to contribution of the wax coverage on flower stems to impeding the locomotion of the generalist ant species *Lasius niger* (Hymenoptera: Formicidae).

However, the reason behind insect attachment force reduction on the petals is less evident. It is plausible to suggest that optimal conditions for attachment of insect pollinators on petals would be advantageous from a plant perspective. That is why the obtained (opposite) result was rather surprising. One possible explanation is related to a need of plants, relying on generalist insect pollinators, to attract latter from a certain distance to their “cafeteria”-type flowers. These individual flowers are usually rather large or, if small, then combined into a rather large inflorescence, as it is the case in the plant species studied here. Many such complex inflorescences (anthodiums) in plants from the family Asteraceae have brightly coloured petals of marginal (ray) flowers. The colour intensity is usually enhanced due to conical or papilla-shaped epidermal cells often supplemented by micro- and nanostructures, such as cuticular folds. It seems to be a result of evolutionary optimization that the enhancement of one function (optical in this particular case), relying on the surface microstructuring, leads to the reduction in the other (mechanical) one. It can be further hypothesized that the function of pollinator attraction is more dominant for “cafeteria”-flowers than providing optimal attachment conditions for pollinators. Since the petals were not particularly large in comparison to the leg span of the syrphid fly studied here, and also to other typical pollinators, such as bees or calliphorid flies, these insects can presumably secure good attachment to the petals by using mechanical interlocking of their tarsal claws to the petal margins without contacting the petal surface with their adhesive pads. Another way, how the insects may avoid problems of low adhesion on *D. pinnata* petals is their tendency to land in the central area of the inflorescence, where the tubular flowers lacking prominent petals are situated.

Additionally, the combination of conical surface structures with cuticular folds found in a number of plant species may be responsible for so called “rose petal effect”, which is based on the ability of certain rough surfaces (with hierarchically organized surface micro- and nanostructures) to have a high contact angle with water, simultaneously with strong adhesion of water (high contact angle hysteresis) [60,61]. This is well-known for petals of plants from, for example, the Rosaceae and Asteraceae families (see Figure 1a) and was even biomimetically implemented in certain technical surfaces [62,63,64]. In plants, this kind of surfaces prevents water from wetting the petals and from rolling drops off the surface into the direction of the flower/inflorescence centre. The surfaces having low wetting ability by water, but stably holding water drops, provides optimal conditions for a quick evaporation of the fluid. Although a thin water film has a high evaporation rate due to its large surface area, breaking such a film in numerous small round droplets, having punctual contacts with the substrate, leads to an even larger overall area that can enhance evaporation rate [60,61,62,63,64]. Moreover, in contrast to the thin water film, this effect supports water evaporation in an almost non-contact state with the petal surface.

Thus, based on the combination of cryo-SEM examination of different surfaces (leaf, flower stem, petal) in the plant *D. pinnata* and the force measurements of the fly *E. tenax* attachment to these surfaces, we showed that the petal surface is not adapted to stronger attachment of generalist insect pollinators. In our opinion, these “cafeteria”-type flowers have the petals, where the colour intensity is enhanced due to papillate epidermal cells covered by cuticular folds at the micro- and nanoscale, and exactly these latter structures mainly contribute to adhesion reduction in insect pollinators. Obtained results may be potentially interesting for fabrication of technical coatings with anti-adhesive properties for insects [52,65,66,67,68,69].

## Data Availability

The data presented in this study are available on request from the corresponding author.
